# Supplementary material for: Predicting heterogeneous ice nucleation with a data-driven approach
Source: Nat Commun. 2020 Sep 22;11:4777. doi: 10.1038/s41467-020-18605-3 (PMC7509812; doi:10.1038/s41467-020-18605-3)
Supplement: Supplementary file 1 — Supplementary Information [file 41467_2020_18605_MOESM1_ESM.pdf]

Supporting Information
Predicting Heterogeneous Ice Nucleation With a Data-Driven Approach

Fitzner et al.

Supplementary Note 1

Additionally to the details mentioned in the main text we provide details on the systems studied. The 900 substrates consist of the following subsets (a more detailed description can be found in Supplementary Table 1):

- OH group patterns (prefix OH_): comprised of oxygen atoms placed in various symmetries on top of a 12-6 Lennard-Jones wall. We employed two wall strengths (0.2 and 0.05 kcal mol^{-1}) and OH patters with triangular, square, hexagonal and octagonal symmetry and combinations thereof. Some of these substrates have been studied in Ref. 1. Oxygen-water interaction was modelled the same way as water-water. Additionally we added the names of the authors as OH group patterns (suffixes _ANGE, _MART and _PHIL), but to our disenchantment found them to be bad nucleators.

- Inorganic fcc substrates (prefix LJ_): The crystalline substrates of Ref. 2, comprising of exposed (111), (100), (110) and (211) surfaces. Distances between atoms and Lennard-Jones interaction between substrate-water were varied to obtain 100 substrates per symmetry.

- Graphitic surfaces (prefix GRA_): Similar to Ref. 3 we use a graphene geometry and vary the (Lennard-Jones) interaction strength.

- Graphene oxide (prefix GRAO_): Taking the graphene geometry, we assign some of the carbons to be oxygens based on a virtual overlay of a rectangular grid of various densities (following Ref. 4). The closes carbon to a virtual grid atom is replaced by an oxygen. The carbon-water interaction is modelled as Lennard-Jones interaction and the oxygen-water interaction is modelled the same as for water-water.

When not described differently we used the following computational settings. The water-water interaction was given by the coarse-grained mW force field.[5] Temperature was maintained by a 10-fold Nosé-Hoover chain[6,7] with a relaxation time of 1 ps. No barostat was

applied. Equations of motion were integrated with a timestep of 10 fs using the LAMMPS[8] software.

Supplementary Figure 1

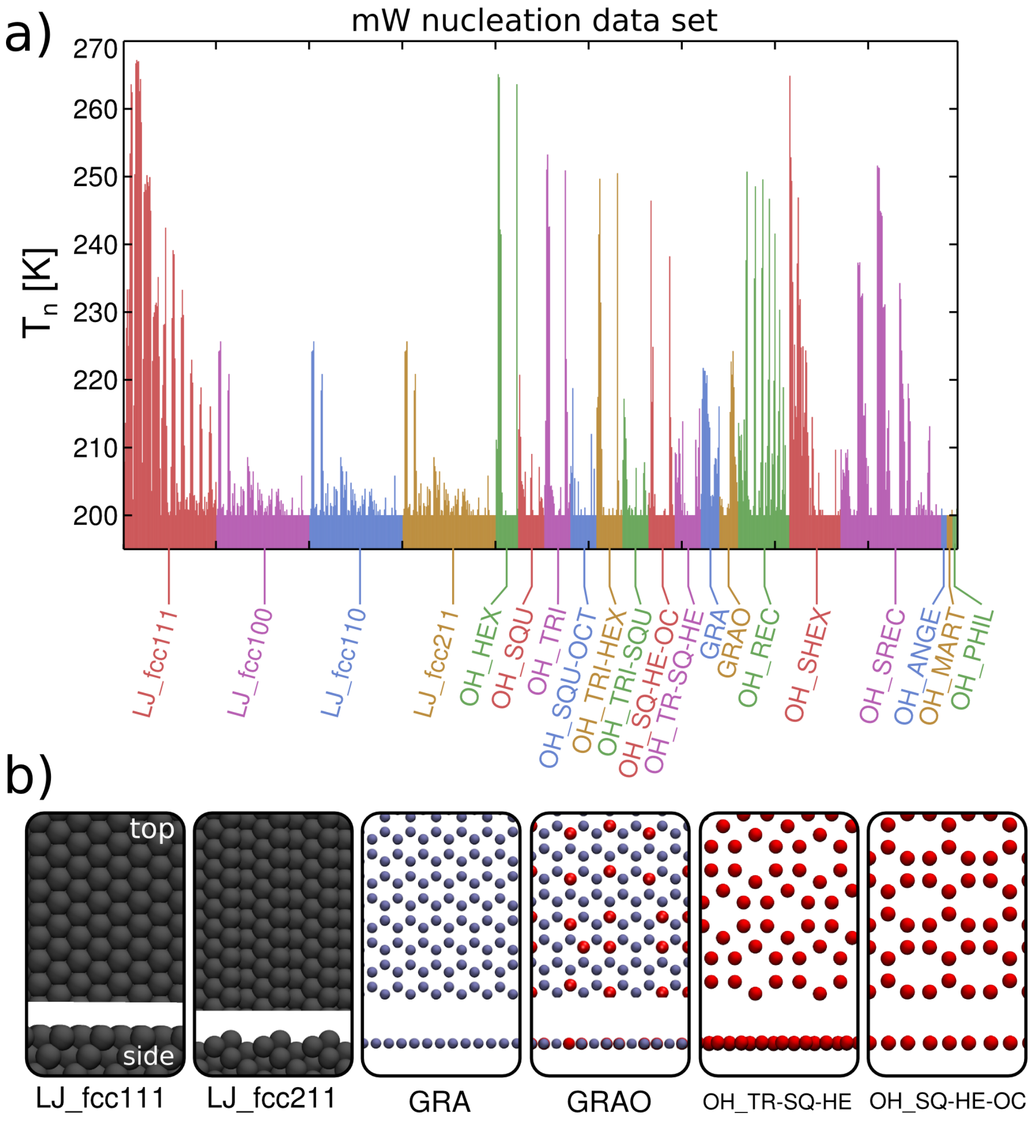

Supplementary Figure 1: a) Distribution of the nucleation temperature T_n sorted by substrate type. b) Exemplary top and side views of different substrate types. LJ atoms are grey, C atoms are blue and oxygens are red.

Supplementary Table 1

Supplementary Table 1: Acronyms of the systems as displayed in Supplementary Figure 1 explained. The interaction of water with the LJ substrates, the back wall and the carbon atoms in graphene were modelled with a Lennard-Jones interaction. The interaction of OH groups with water was treated as mW-mW[5] interaction.

Acronym	Description	Interaction	Ref.
LJ_fcc111	Fcc crystal with (111) surface exposed	LJ	2
LJ_fcc100	Fcc crystal with (100) surface exposed	LJ	2
LJ_fcc110	Fcc crystal with (110) surface exposed	LJ	2
LJ_fcc211	Fcc crystal with (211) surface exposed	LJ	2
OH_HEX	Back wall + hexagonal OH patterns	LJ + mW	1
OH_SQU	Back wall + square OH patterns	LJ + mW	1
OH_TRI	Back wall + triangular OH patterns	LJ + mW	1
OH_SQU-OCT	Back wall + OH squares and octagons	LJ + mW	1
OH_TRI-HEX	Back wall + OH triangles and hexagons	LJ + mW	1
OH_TRI-SQU	Back wall + OH triangles and squares	LJ + mW	1
OH_SQ-HE-OCT	Back wall + OH squares, hexagons and octagons	LJ + mW	1
OH_TR-SQ-HE	Back wall + triangles, squares and hexagons	LJ + mW	1
GRA	Graphene structure of varied interaction strength	LJ	3
GRAO	Graphene structure + OH patterns	LJ + mW	4
OH_REC	Back wall + rectangular OH patterns	LJ + mW	-
OH_SHEX	Back wall + stretched hexagonal OH patterns	LJ + mW	-
OH_SREC	Back wall + stretched rectangular OH patterns	LJ + mW	-
OH_ANGE	Name of the author as OH pattern	LJ + mW	-
OH_MART	Name of the author as OH pattern	LJ + mW	-
OH_PHIL	Name of the author as OH pattern	LJ + mW	-

Supplementary Note 2

We give a brief overview of the initial features we consider an how they were computed. Supplementary Figure 2 shows an illustration of the different feature classes and how the corresponding acronym is formed. Each graph starting from a blue box can be considered a new class of features which we then pre-process to obtain statistical measures (*stat* in red in Supplementary Figure 2) for the corresponding quantities while also distinguishing different layers perpendicular to the surface (*layers* in green in Supplementary Figure 2, see also the inset on the top right) for some of them. Features are organized in different families as follows:

- *dyn*:

  Starting point is a simulation of liquid water interfacing with the substrate. We run two sets, one which is 100 ns long where we save every 1 ps (for Steinhardt q_l,[9] local Steinhardt lq_l[10] and number of nearest neighbors *nn*) and one which is 100 ps where we save every 10 fs (for forces and velocities).

- *disp*:

  Displacements in either dimension (x, y, z, lateral xy and total r) after 1, 2, 5, 10, 20, 35, 50, 75, 100 and 150 ps.

- *rssA*:

  Random structure search approach similar to Ref. 11 where we probe the adsorption energy of hemispherical ice seeds ($I_h(001)$, $I_h(100)$, $I_h(110)$, $I_c(001)$ and $I_c(111)$) for different sizes (100, 300 and 500 molecules).

- *rssB_flex*:

  Energies from minimization of n-mer water clusters and cages positioned in many random positions above the surface. The ice structures are free to relax and adjust.

- *rssB_rigid*:

Similar to the flexible approach but keeping the structure of the deposited ice structure rigid. Since energy minimization with rigid bodies is highly not-trivial we performed short MD simulations with rigid constraints,[12,13] slightly pushing the ice-structure downwards while draining out the kinetic energy with a friction term in the equations of motion. In this manner we find that the ice structures have enough room to find a local minima with respect to position and orientation.

- *lmatch*:

  Generalized lattice match calculated as in Ref.:[1]

$$\zeta = \min_{\mathbf{r}_0,\theta} \left(\sqrt{\frac{1}{N_\mathrm{M}} \sum_{i=1}^{N_\mathrm{ice}} \left(\mathbf{r}_i(\mathbf{r}_0,\theta) - \mathbf{r}_\mathrm{s}\right)^2} \right) \tag{1}$$

  Here we place ice layers corresponding to a certain face randomly over the surface and compute the shortest distance to a substrate atom for each ice molecule provided this is shorter than a certain cutoff (yielding N_M contacts). We considered 2D projections of the ice lattices as well as their actual 3D structure and different cutoffs for neighbor choices. Ice faces considered were $\mathrm{I_h}(001)$, $\mathrm{I_h}(100)$, $\mathrm{I_h}(110)$ and $\mathrm{I_c}(001)$.

- *dens*:

  Number density of liquid water in different layers obtained from the dyn runs.

Here are four examples of acronyms and their actual meaning:

1. **lmatch2D_Ih001_c2**:

   Generalized lattice match calculated with the second cutoff (c2 = 3.2 Å) of the 2D projected basal face (Ih001)

2. **dyn_nn_all_median**:

   Median number of nearest neighbors in the whole liquid (cutoff 3.4 Å)

3. **rssBr_4mer_Eall_range**:

Total range of all adsorption energies of the water tetramer obtained with the rigid random structure search approach

4. **dyn_lq3_l12_var**:

   Variance of the lq_3 parameter in the water layer l12 (definition in Supplementary Figure 2)

Supplementary Figure 2

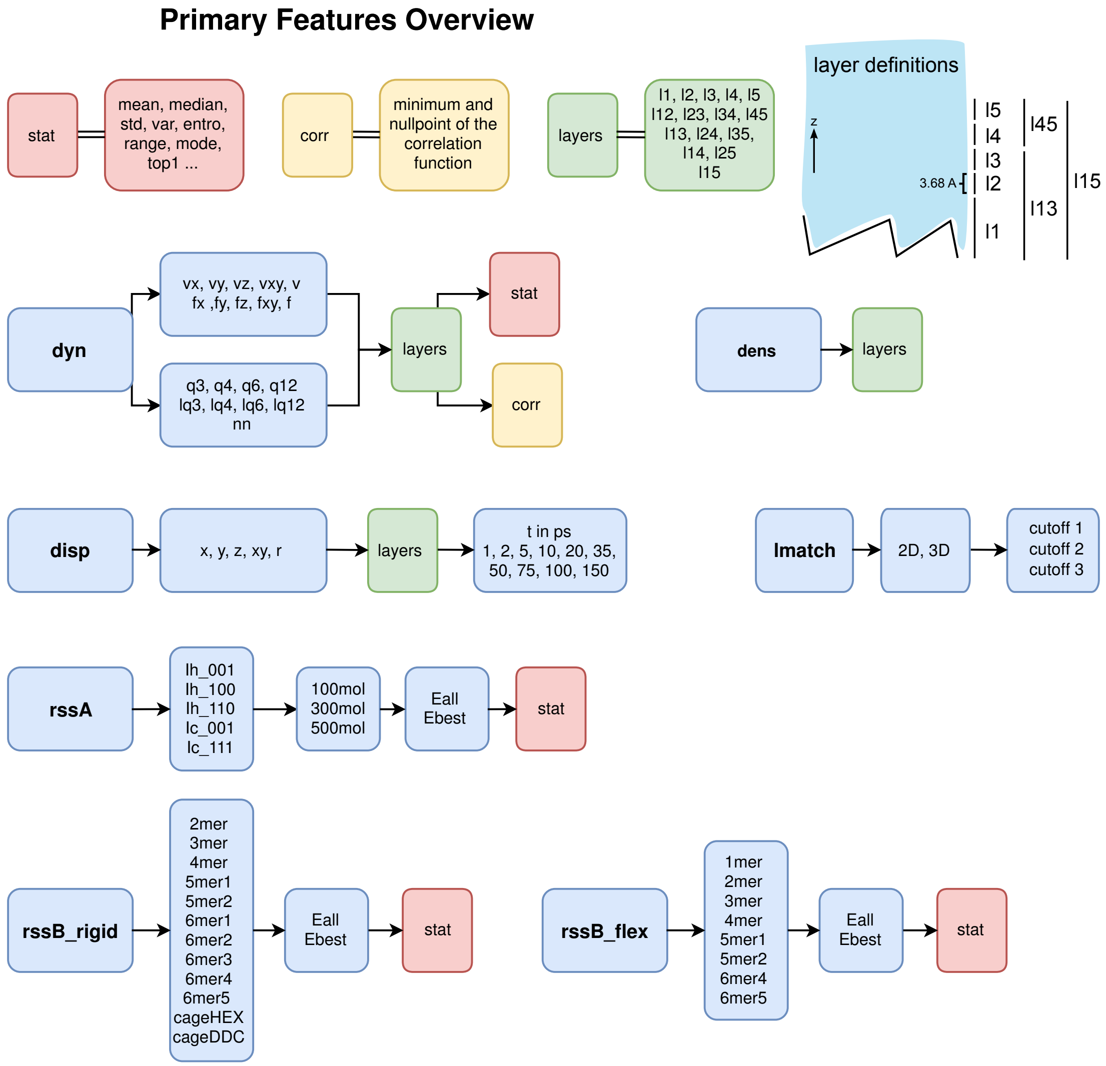

Supplementary Figure 2: Overview of the different feature families and their automatic namings. The meanings of “dyn”, “disp”, “rssA”, “rssB_rigid”, “rssB_flex”, “dens” and “lmatch” are explained in the text. Names are created by following the arrows, e.g. “disp_xy_l3_50” or “dyn_lq3_l1_mean” are possible names describing “the xy-displacement in the 3rd layer after 50ps” and “the mean lq3 vaues in the first layer” respectively. The illustration on the top right shows the definition of layers, where each single layer has a z-extension of 3.68Å.

Supplementary Note 3

We provide a few more details on the algorithms and metrics used in the machine learning workflow.

After the cooling ramps are performed and after all possible features have been computed we train a random forest model.[14] To this end we specifying a substrate as good when the average T_n was > 225 K and as bad otherwise. The model is trained to predict whether a substrate is good or bad, which is a binary classification problem.

A random forest is a collection of decision trees. A single decision tree can be created by performing binary splits regarding certain variables, where in each step the variable is chosen that gives the best improvement in the training metric. This is done until a maximum number of splits is reached or if the metric improvement is below a certain threshold. The random forest is now created by fitting many such decision trees, but randomly selecting a subset of all data for each tree. The substrates are selected via bootstrapping but also the features used in each decision tree are a subset of all possible features, randomly selected to equal around $\sqrt{N}$, where N is the total number of features.

As training metric we choose the gini index $1 - \sum_i (p_i)^2$, where p_i are the probabilities of being classified as class i (lower is better). Since the metric is evaluated on out-of-bag samples (i.e. the ones that are not used for training that particular tree) there is little danger of overtraining the random forest. We train the forest several times with 10000 trees and gather the mean feature importance of all features. The feature importance is calculated by randomly permuting the values of a feature and comparing the decrease in the performance metric compared to the unpermuted case. We additionally choose to restrict this evaluation to the performance metric calculated to the class of good nucleators to get the most important features for being able to tell what is a good nucleator.

To deal with feature correlations we cluster the features by hierarchical clustering, a classical and simple clustering algorithm which relies on a distance metric. We want to consider two features f_1 and f_2 as close when they are strongly correlated. Thus, we define

the distance as $d(f_1, f_2) = 1 - \text{MIC}(f_1, f_2)$, where $\text{MIC}(f_1, f_2)$ is the maximum information coefficient.[15] The MIC is essentially a variant of mutual entropy that measures common information in two features, additionally screens through many different grid sizes that are needed to calculate the necessary histograms, and is also bound between 0 and 1. Most importantly (and contrary to standard Pearson correlation) it is able to recognize non-linear and periodic correlations and thus suited very well for our distance metric.

The clustering algorithm works in a simple manner. It starts by assigning two two closes data points to a cluster. This is repeated until all possible components are connected. After data points are assigned to a cluster, this cluster is regarded as new data point, and distances to this new point are calculated in different ways. When a new distance to that cluster should be calculated we take the mean distance to all the data points in that cluster. The choice of this is called average linkage. We also tested other feature selection approaches that are not based on clustering, see Supplementary Note 4.

The feature clustering allows for a systematic identification of n clusters, in that connected components with the largest distance are iteratively cut until n clusters are left. This can be used to select n features by generating n clusters in this manner and selecting out of each cluster the feature with the highest importance. The features selected in such a way are to some degree decorrelated, but also important for the prediction task (in our case to the target variable T_n). Supplementary Figure 4 shows a matrix with MIC correlations between features that is ordered by the feature distances together with several choices of how n clusters would be selected.

We have tried several models for training the prediction task for T_n. We treat this problem as a regression problem, i.e. the exact value of T_n should be predicted. We have used random forest,[14] a XGBoost[16] and support vector machines.[17,18] The former was explained in detail in the previous subsection. XGBoost is a popular variant for gradient boosting with trees. It is also based on single decision trees, however the model is not averaging the votes of all trees like in a random forest. Rather, the model $\hat{y}_i$ is built iteratively, adding new trees $\hat{t}$ that

are fit to predict the residual error between the previous model and the prediction target y: $\hat{y}_i = \hat{y}_{i-1} + \eta \cdot \hat{t}_i = \sum_k^i \eta \cdot \hat{t}_k$, where η is the so called learning rate. In this manner the model learns how to correct its own errors. The support vector machine uses a kernel to map points into a space in which they a separable in a manner corresponding to the separation in the target variable. For the details the reader is referred to the literature.

All models come with a variety of hyperparameters, for instance the number of trees to use for a random forest or the learning rate for boosting. We are using a Bayesian tree-structured method from the pyhton package hyperopt[19] to guide the search for the optimal hyperparameters in all cases allowing for 200 search iterations. This search is done in the inner cross-validation loop and the best found hyperparameters are used to evaluate on the test set.

Supplementary Figure 3

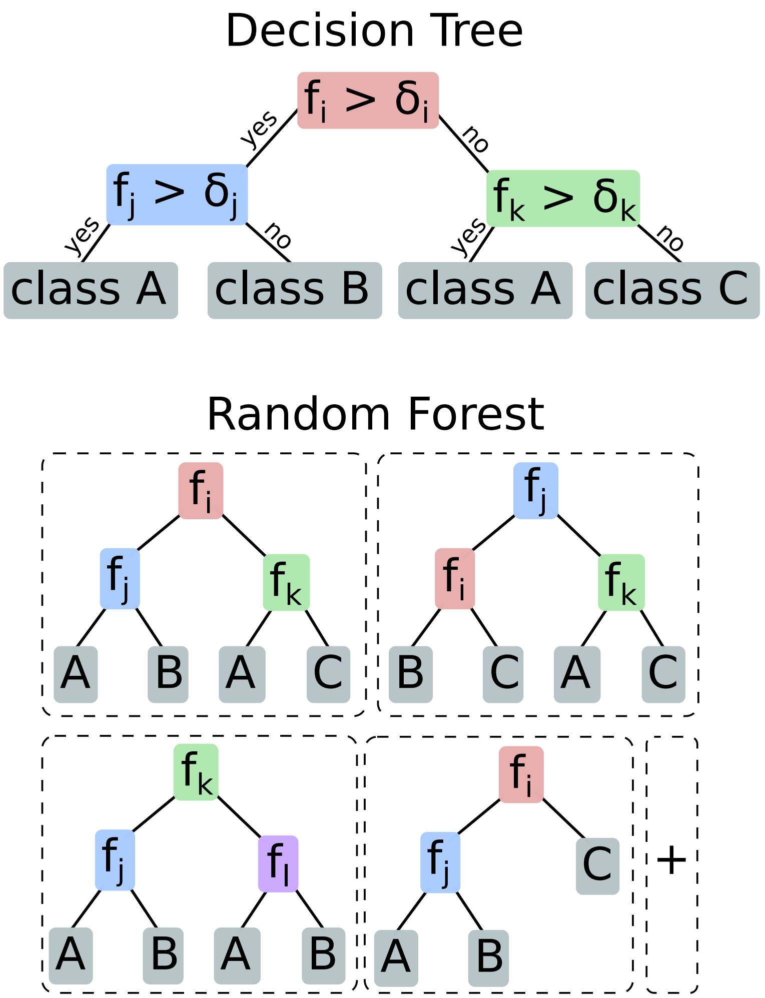

Supplementary Figure 3: Illustration of random forest models consisting of single decision trees for a classification problem. f_i are possible features that are split on thresholds δ_i.

Supplementary Figure 4

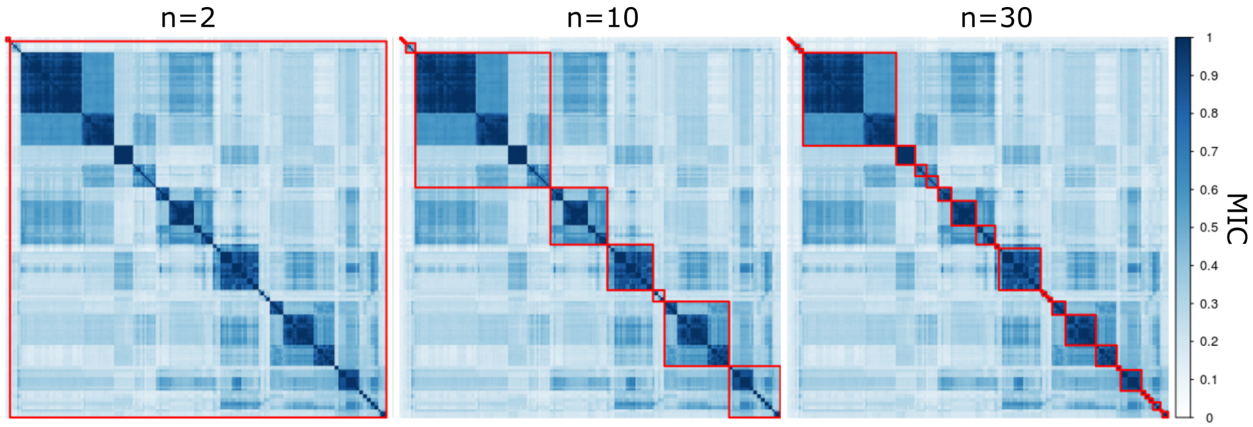

Supplementary Figure 4: Feature correlation matrix. Red squares correspond to clusters formed when forming $n = 2, 10, 30$ clusters (from left to right).

Supplementary Note 4

In Supplementary Figure 5 we show the feature selection results for different methods. We can see that the results differ slightly. But the differences are not major and remarkably, there are families of features that appear frequent.

Besides the clustering method described earlier to select features (here referred to as method 1) we have also tried two other methods. Method two iteratively selects features by descending importance, if the mean MIC with already selected features is below 0.4. Method 3 is the same but checks the maximum MIC rather than the mean. We find that results can differ (see Supplementary Figure 5), especially for the less important features. Overall, similar choices of particular features are made and most importantly, the selected features are from similar families, which demonstrates a degree of consistency.

Supplementary Figure 5

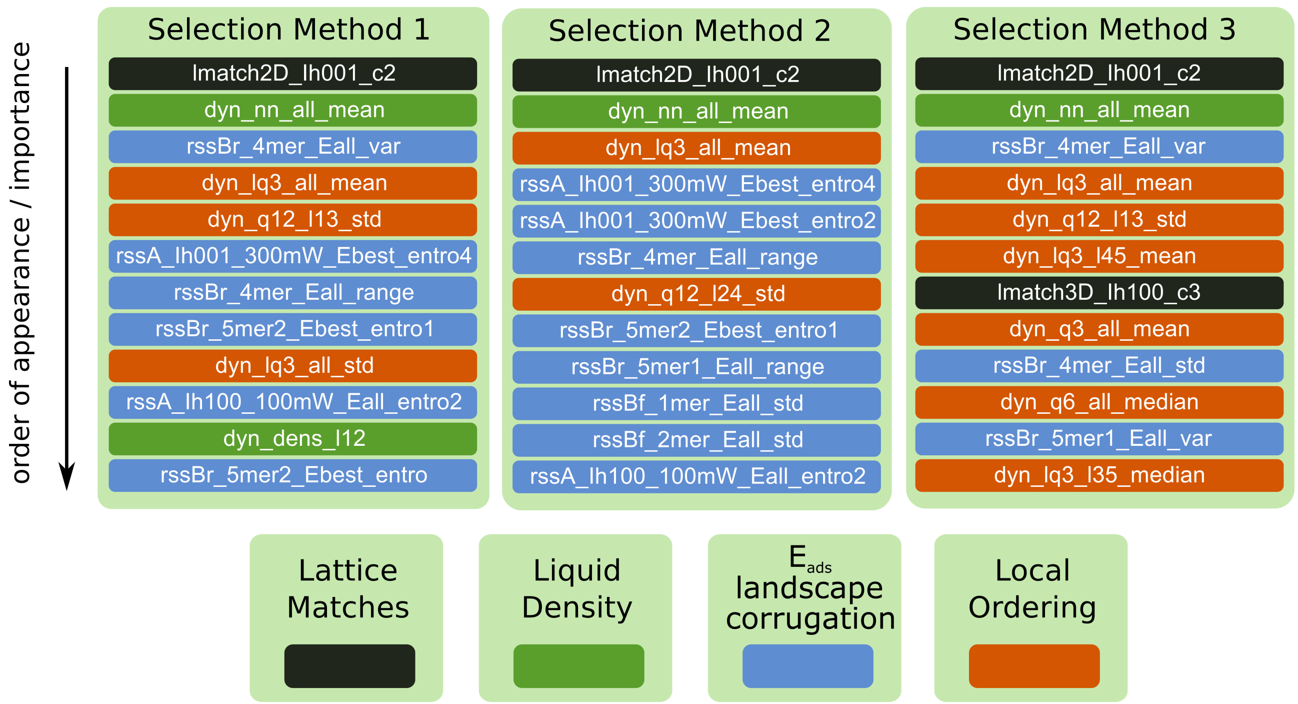

Supplementary Figure 5: Selected features for the three tested feature selection methods. Features are ordered by selection appearance from top to bottom. Tile background colors correspond to feature families as indicated on the bottom.

Supplementary Note 5

In the main text we have assessed the model performance on a regression problem. We have also probed a classification problem and show the results in Supplementary Figure 6. We split the substrates in good and bad nucleators (T_n threshold at 225 K) and calculated the F1 score. The F1 is the harmonic average of precision and recall and thus is less susceptible to class imbalance. Generally, values above 0.8 are considered good and values above 0.9 are considered excellent. The baseline model we compare to is the 5-nearest neighbor classification. As we can see we can achieve almost excellent results if the first four features are included which reaffirms the finding for the regression task. The classification problem is also easier since we are closer to the perfect score and also the baseline model does not perform as badly as for the regression.

Supplementary Figure 6

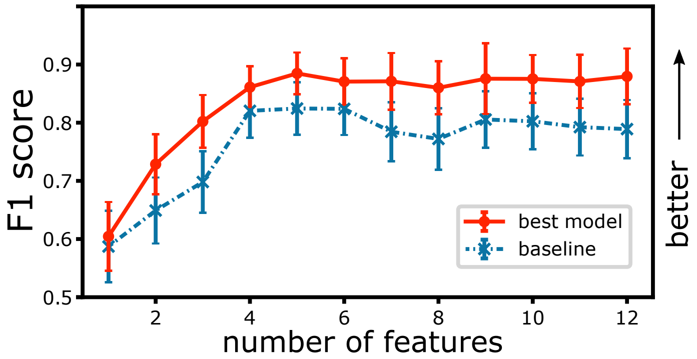

Supplementary Figure 6: Model performance for the classification problem of identifying good and bad nucleators (split by T_n value at 225 K). Source data are provided as a Source Data file.

Supplementary Note 6

It is beyond the scope of this work to benchmark many different machine learning models, however a brief assessment was done in order to get a feeling of the difficulty of the task. We find that random forest and XGBoost perform best (the latter is discussed in the main text) and also considerably better than the mean guess and linear model (elastic net). The trend of decreased improvement after 4 included features is also clear.

Supplementary Figure 7

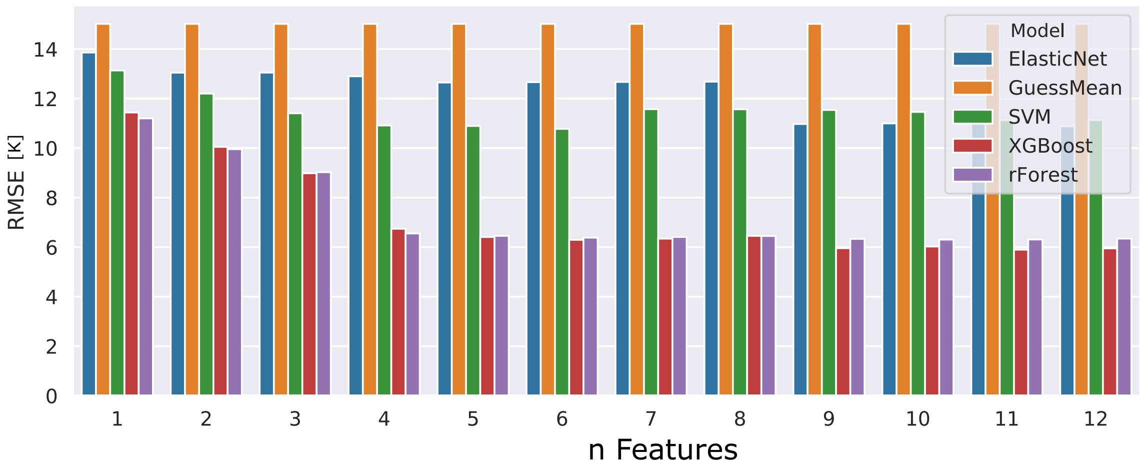

Supplementary Figure 7: RMSE of different machine learning models as a function of the number of features included. We compare a linear elasic net (ElasticNet), the mean-guess for T_{n} (GuessMean), a support vector machine with radial kernel (SVM), XGBoost and a random forest (rForest). Search for hyperparameters was done with hyperopt[19] and 200 iterations. Source data are provided as a Source Data file.

Supplementary Note 7

More evidence that the descriptors identified in this work are capable of distinguishing good and bad nucleators can be seen in Supplementary Figure 8. In there we show a linear (PCA) and non-linear (ISOMAP) dimensionality reduction plot of the 9 features shown in Supplementary Figure 9.

Supplementary Figure 8

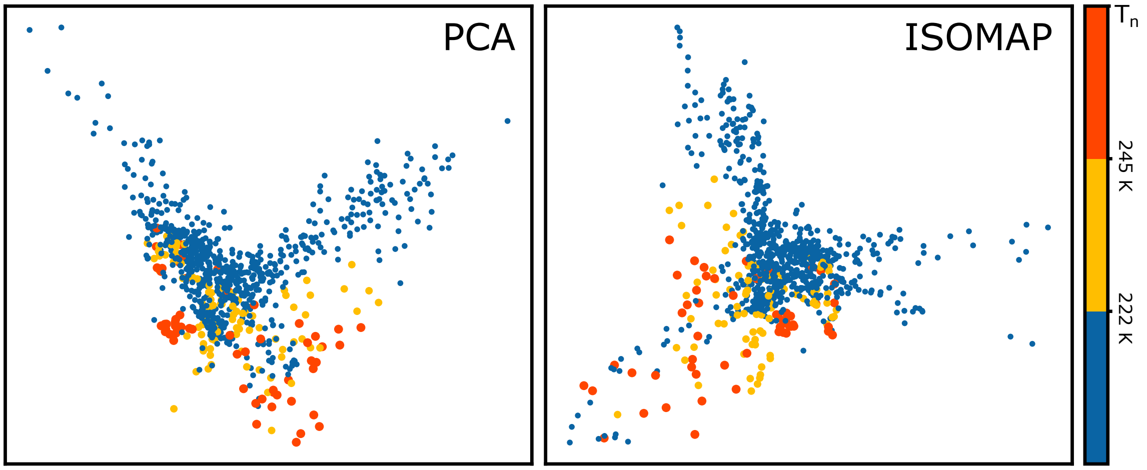

Supplementary Figure 8: Dimensionality reduction plots for all substrates using the 9 features shown in Supplementary Figure 9. Shown are the first two components. Points are colored by their T_n where we split the data into three classes as indicated by the color bar on the right.

Supplementary Note 8

We provide the SHAP value[20] distributions of a few more features in Supplementary Figure 9. They are ordered by mean SHAP value impact and while that order generally follows the trend from Supplementary Figure 5 it does not necessarily lead to the exact same order.

Supplementary Figure 9

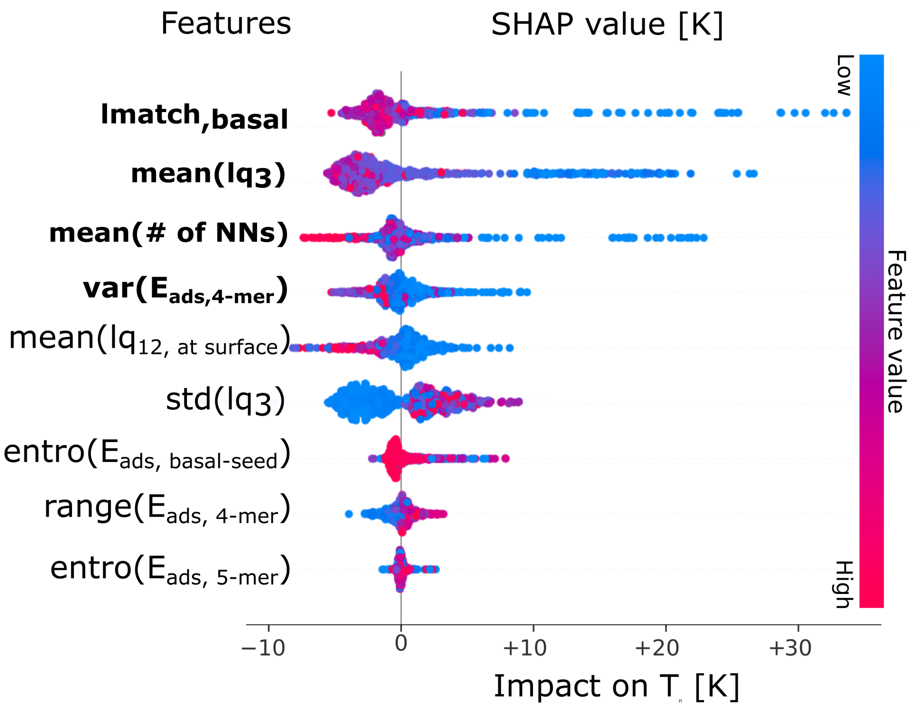

Supplementary Figure 9: SHAP value distribution for the first 9 features identified with the cluster-based feature selection method. Bold entries are discussed in the main text.

Supplementary References

(1) Pedevilla, P.; Fitzner, M.; Michaelides, A. What makes a good descriptor for heterogeneous ice nucleation on OH-patterned surfaces. *Phys. Rev. B* **2017**, *96*, 115441.

(2) Fitzner, M.; Sosso, G. C.; Cox, S. J.; Michaelides, A. The Many Faces of Heterogeneous Ice Nucleation: Interplay Between Surface Morphology and Hydrophobicity. *J. Am. Chem. Soc.* **2015**, *137*, 13658–13669.

(3) Cox, S. J.; Kathmann, S. M.; Slater, B.; Michaelides, A. Molecular simulations of heterogeneous ice nucleation. II. Peeling back the layers. *J. Chem. Phys.* **2015**, *142*, 184705.

(4) Lupi, L.; Molinero, V. Does Hydrophilicity of Carbon Particles Improve Their Ice Nucleation Ability? *J. Phys. Chem. A* **2014**, *118*, 7330–7337.

(5) Molinero, V.; Moore, E. B. Water Modeled As an Intermediate Element between Carbon and Silicon. *J. Phys. Chem. B* **2009**, *113*, 4008–4016.

(6) Martyna, G. J.; Klein, M. L.; Tuckerman, M. Nosé-Hoover chains: The canonical ensemble via continuous dynamics. *J. Chem. Phys.* **1992**, *97*, 2635–2643.

(7) Nosé, S. A unified formulation of the constant temperature molecular dynamics methods. *J. Chem. Phys.* **1984**, *81*, 511–519.

(8) Plimpton, S. Fast Parallel Algorithms for Short-Range Molecular Dynamics. *J. Comput. Phys.* **1995**, *117*, 1–19.

(9) Steinhardt, P. J.; Nelson, D. R.; Ronchetti, M. Bond-orientational order in liquids and glasses. *Phys. Rev. B* **1983**, *28*, 784–805.

(10) Li, T.; Donadio, D.; Russo, G.; Galli, G. Homogeneous ice nucleation from supercooled water. *Phys. Chem. Chem. Phys.* **2011**, *13*, 19807–19813.

(11) Pedevilla, P.; Fitzner, M.; Sosso, G. C.; Michaelides, A. Heterogeneous seeded molecular dynamics as a tool to probe the ice nucleating ability of crystalline surfaces. *J. Chem. Phys.* **2018**, *149*, 072327.

(12) Miller III, T.; Eleftheriou, M.; Pattnaik, P.; Ndirango, A.; Newns, D.; Martyna, G. Symplectic quaternion scheme for biophysical molecular dynamics. *J. Chem. Phys.* **2002**, *116*, 8649–8659.

(13) Zhang, Z.; Glotzer, S. C. Self-assembly of patchy particles. *Nano Lett.* **2004**, *4*, 1407–1413.

(14) Breiman, L. Random forests. *Machine learning* **2001**, *45*, 5–32.

(15) Reshef, D. N.; Reshef, Y. A.; Finucane, H. K.; Grossman, S. R.; McVean, G.; Turnbaugh, P. J.; Lander, E. S.; Mitzenmacher, M.; Sabeti, P. C. Detecting novel associations in large data sets. *Science* **2011**, *334*, 1518–1524.

(16) Chen, T.; Guestrin, C. Xgboost: A scalable tree boosting system. Proceedings of the 22nd ACM SIGKDD International Conference on Knowledge Discovery and Data Mining. 2016; pp 785–794.

(17) Cortes, C.; Vapnik, V. Support-vector networks. *Mach. Learn.* **1995**, *20*, 273–297.

(18) Ben-Hur, A.; Horn, D.; Siegelmann, H. T.; Vapnik, V. Support vector clustering. *J. Mach. Learn. Res.* **2001**, *2*, 125–137.

(19) Bergstra, J. S.; Bardenet, R.; Bengio, Y.; Kégl, B. Algorithms for hyper-parameter optimization. Advances in Neural Information Processing Systems. 2011; pp 2546–2554.

(20) Lundberg, S. M.; Lee, S.-I. A unified approach to interpreting model predictions. Advances in Neural Information Processing Systems. 2017; pp 4765–4774.
